# Direct Flexor Tendon Repair More than 3 Months After Trauma: Clinical Outcomes of Four Consecutive Cases and Scoping Review on Time Limits

**DOI:** 10.3390/jcm14165796

**Published:** 2025-08-16

**Authors:** Alessandro Crosio, Alice Clemente, Arturo Sebastiano Nozzolillo, Sara Dimartino, Simona Odella, Davide Ciclamini, Pierluigi Tos

**Affiliations:** 1 UOC Chirurgia Della Mano E Microchirurgia Ricostruttiva, Asst Gaetano Pini-Cto Milano, Piazza Cardinale Ferrari, 1, 20122 Milan, Italy; dr.clementealice@gmail.com (A.C.); arturosnozzolillo@gmail.com (A.S.N.); simona.odella@asst-pini-cto.it (S.O.); 2 SSD Microchirurgia Ortopedico Traumatologica, Ortopedia e Traumatologia 2, Chirurgia Della Mano, AOU Città Della Salute E Della Scienza—Po CTO, Via Zuretti 29, 10126 Torino, Italy; 3 SC Ortopedia, AOU Policlinico “G. Rodolico—San Marco”, PO San Marco, Piazzale C.A. Ciampi, 95121 Catania, Italy; saradimartino1@gmail.com; 4 Ortopedia e Traumatologia, AOU Ospedale Maggiore Della Carità, Corso Mazzini 18, 28100 Novara, Italy; davide.ciclamini@gmail.com

**Keywords:** flexor tendon, delayed primary repair, hand therapy, tendon repair, hand surgery, scoping review

## Abstract

**Background/Objective:** Traumatic injuries to the flexor tendons of the hand are frequently treated by hand surgeons. Late repair is not classically considered to be feasible due to the high risk of failure and functional complications. The present study aims to present the functional results of primary flexor tendon repairs performed more than three months after trauma, along with evidence regarding the time limit for primary flexor tendon repair. **Methods:** The clinical outcomes of direct flexor tendon repairs in zones 1 and 2 of the long fingers or thumb are reported herein. A scoping review was undertaken using Medline and CINHAL to identify studies reporting the functional outcomes of flexor repair following trauma. **Results:** In this series, four patients were treated with direct M-Tang and epitendinous suture or pull-out reinsertion. Accessory procedures were required to perform a direct repair. The mean delay was 5.5 months, and the follow-up period was 24 months. The mean total active movement was 195°. Extension lags of 10° and 20° were registered at the proximal interphalangeal and distal interphalangeal joints, respectively. While a literature review showed that most cases treated with primary repair after three months resulted in functional complications, these procedures were performed around 40 years ago and no recent reports were found. **Conclusions:** In the small cohort of patients here reported it has been possible to repair flexor tendons in zones 1 and 2, and to reinsert a jersey finger, even three months after trauma. Accessory procedures were required. Accurate patient selection and counseling is mandatory before surgery to inform patients about alternatives. The literature review confirmed that no positive results have previously been reported in the literature on this topic. It is thought that modern materials and surgical techniques for flexor tendon repair should extend the edge for primary repair in selected patients, as compared to previous practices.

## 1. Introduction

Traumatic injuries of the hand flexor tendons refer to acute mechanical disruptions of the flexor tendon structures in the long fingers or thumb. They can present as open injuries, typically caused by penetrating or sharp forces. Closed tear injuries often involve the flexor digitorum profundus (FDP), leading to avulsion of the tendon from its bony insertion, with various types of avulsion patterns described.

Hand flexor tendon traumatic injuries represent events that are frequently observed by hand surgeons. The incidence of such injuries is around 5.4 cases per 100,000 people each year [1].

Despite significant advances in knowledge of the pathophysiology of tendinous injuries, improvements in the surgical techniques available to modern hand surgery and the development of targeted rehabilitation protocols, such injuries still represent conditions requiring complex orthopedic management and are often burdened by suboptimal functional outcomes. The traumatic mechanism, the patient’s age and comorbidities, the presence of associated bony or nerve injuries, the extent of soft tissue damage, and the surgeon’s experience represent the main factors related to the occurrence of postoperative complications [2].

There is no definitive evidence in the literature regarding the ideal time to perform tendon repair. Traditionally, prompt surgical intervention within 24 to 72 h following traumatic flexor tendon injury was regarded as essential to achieve optimal functional outcomes [3,4,5,6,7,8,9,10,11,12,13,14,15,16]. Nonetheless, recent literature indicates that satisfactory results can still be obtained when repairs are performed within seven days [17,18]. Emerging data and surgeon consensus derived from case series propose an extended acceptable window ranging from five to six weeks [19,20]. A survey of 91 international hand surgeons revealed a division in opinion: 34% identified three weeks as the upper limit for primary repair, 32% favored four weeks, and 36% supported five weeks as a reasonable threshold [4]. Ejeskär previously suggested a maximum limit of three months, beyond which primary repair was deemed unlikely to restore function [3]. Across the spectrum of studies, delayed primary repair up to approximately six weeks remains viable; however, procedures beyond this timeframe are typically discouraged due to progressive soft tissue changes such as adhesion formation, muscular shortening, and collapse of the pulley system [3,4,5,18,19,20].

In this retrospective cohort study, functional outcomes of patients treated with primary flexor tendon repair or reinsertion beyond the previously proposed time frame are presented, together with a discussion of the indications and surgical techniques adopted. In addition, a scoping review was conducted to examine the management of primary flexor tendon repair in very late presentations, assessing the relevance of the proposed time frame and summarizing the solutions and outcomes reported in the literature.

## 2. Materials and Methods

The authors conducted a retrospective evaluation of functional outcomes for patients who had undergone deep flexor tenorrhaphy and deep flexor reinsertion. Only patients with tendon injuries in zones 1 and 2 according to Verdan and jersey finger type 1 treated at least 3 months after injury between March 2021 and March 2022 were included [6]. A functional evaluation was performed 24 months after surgery, when the Total Active Motion (TAM) was calculated, as described by the American Society for Surgery of the Hand (ASSH) [7].

In parallel, a scoping review of the literature was performed to investigate the functional outcomes of the longest-delayed primary flexor tendon repairs in the hand in adults. The inclusion criteria were primary repair, in the English or French literature, and limited to adults. The concept was the time from injury as a limit for primary repair and functional outcomes. The context was primary repair of flexor tendon injuries. The research took place on Medline and CINHAL as follows: flexor tendon delayed repair, no time limit set. The title, abstract, and text were analyzed by the authors according to the inclusion criteria. The selected papers were included in tables summarizing the “intervention type”, “population”, “duration of intervention”, “aims”, “methodology adopted”, “key findings”, and “gaps in the research”.

### 2.1. Surgical Technique

All patients underwent thorough preoperative counseling, including discussion of all surgical options and the possibility of final intraoperative decision-making. Rehabilitation protocols and expected timelines for functional recovery were also explained.

Direct repair was performed when the gap, with the finger in extension and A2/A4 pulleys intact, did not exceed 3 cm. Procedures were undertaken by surgeons with Tang and Giddins level 3–4 experience and under axillary plexus anesthesia [8]. Incisions incorporated previous scars when present, and were extended via midlateral or Brunner approaches, as appropriate. The pulley system and distal tendon stump were identified, usually by opening the A3 pulley; in one case, the proximal stump was found beneath the A2. The pulley system was explored with a beaver blade to confirm sufficient passage space.

For 1 cm gaps (wrist neutral, PIP slightly flexed), partial transection of the FDP5 musculotendinous junction was performed as per Le Viet [9]. Stumps were repaired in neutral position with appropriate digital cascade tension, using a 6-strand M-Tang core suture reinforced by a Silfverskiöld epitendinous stitch. Suture integrity was confirmed with an extension test and passive wrist flexion.

For a 2 cm gap (FPL injury), Z-lengthening as per Vigliani et al. [10] was performed to gain up to 3 cm, avoiding tendon grafting.

In the type 1 jersey finger case, the proximal stump was retrieved at the MP joint, followed by extensive palm and wrist tenolysis to advance the stump to P3 and complete reinsertion with the pull-out technique.

The A2 pulley was preserved in all cases; A4 venting was performed when necessary to prevent suture impingement. One patient underwent ulnar collateral nerve gap reconstruction with a muscle-in-vein graft.

### 2.2. Postoperative Protocol

At the end of the procedure, a dorsal intrinsic plus splint was placed with the wrist in neutral, metacarpophalangeal joints (MPj) flexed at 45°, and the interphalangeal joints (IPj) extended. Beginning on the 3rd–4th postoperative day, consistent with the patient’s pain level, a physiotherapist-assisted early active motion rehabilitation protocol was activated. In particular, mid-range active motion was started immediately, and the patients were instructed to perform passive and active exercise at home 3 times a day. A clinical check of the surgical wound was performed weekly. The splint was discontinued in postoperative week 5. The active motion was increased to complete flexion from week 5 to week 8. Restriction of heavy activity was continued up to 3 months after surgery. For the present study, the following data were collected regarding the patients treated: age, sex, date of trauma, time interval between injury and surgical repair, injured finger and any associated injuries, and follow-up time. At a follow-up visit 2 years after surgery, the range of motion of the metacarpophalangeal (MP), proximal interphalangeal (PIP), and distal interphalangeal (DIP) joints was assessed using a goniometer. For thumb evaluations, the Kapandji score was also recorded. The Total Active Motion (TAM) was then calculated, as described by the American Society for Surgery of the Hand (ASSH), as the sum of the degrees of active flexion of the MP, PIP, and DIP minus the degrees of flexion from maximum extension [7].

## 3. Results

### 3.1. Retrospective Evaluation

Four patients met the inclusion criteria: two flexor digitorum profundus injuries of the little finger, one flexor pollicis longus injury of the thumb, and one jersey finger type 1 injury of the ring finger. The patients’ data are shown in Table 1.

The delay between the traumatic event and the surgical procedure was between 3 and 8 months (mean 5.5 months).

The age of the patients ranged from 19 to 34 years (mean 29 years and 9 months).

Each of the injuries was caused by a knife, metal sheet, or glass fragments.

Associated injuries were represented by an ulnar collateral nerve injury of the little finger in one case and a fracture of the head of the middle phalanx in the ring finger in another case. As stated above, the collateral nerve injury was repaired by means of a muscle-in-vein combined graft, and this did not affect the postoperative protocol. Similarly, the composed fracture of P2 in one patient did not interfere with recovery.

The patients were followed up at months 1, 2, 3, 6, and 12 in outpatient services, and the final follow-up with inclusion in this report was at 24 months. The mean TAM value obtained was 195° (range 135–240°) (Table 2).

The extension lag for the DIP was of 10° in one case and 20° in another.

The percentage value of TAM obtained by each patient compared with the normal value (equal to 260° for the long fingers and 180° for the thumb) was then calculated. By applying the ASSH scheme regarding TAM percentages, the following results were obtained: an “excellent” result was obtained in one patient, a “good” result in two patients, and an “acceptable” result in one patient (Table 2). In the case where the FPL was repaired, the Kapandji score reached 9 over 10.

There were no cases of suture rupture or cases of intolerance to the suture threads that were used. Photos and videos of the four patients taken at the last checkup are available (Supplementary Figures S1–S4 and Supplementary Videos S1–S4).

### 3.2. Scoping Review Results

**Figure 1:**
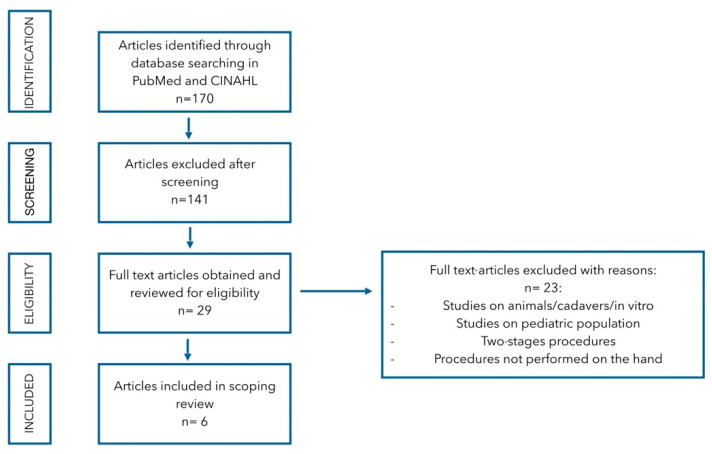
Flow diagram of literature search and study selection.

A total of 170 papers were identified. After title screening, 29 works were considered; after abstract evaluations, only 6 papers were finally included. The scheme of the review is summarized in Figure 1.

In Table 3, the main aspects of each paper are summarized. Only one patient was treated with direct reinsertion of the FDP due to a jersey finger injury. The delay between trauma and treatment was 6 weeks, and Z-lengthening at the musculotendinous junction was performed to complete a direct suture [11].

Regarding flexor tendon cut injuries, 128 patients were included in this review. Few of them were treated past the 5-week mark. Suzuki reported three children receiving delayed direct repair without any ancillary procedure other than fascial graft [12]. Ejeskär reported numerous patients treated after 3 months [3]. In their case series, most of the delayed patients presented complications such as extension lag or secondary rupture. It was not specified what delayed repair meant in these cases, since the injuries were repaired between 2 and 14 weeks after trauma. Munz treated all patients within 5 weeks of injury, finding good recovery without ancillary procedures [19]. The five patients treated by Durand were all treated after a delay of 5 months [13]. Durand used an emi-FDP transfer from an adjacent finger to repair the injury directly. He did not attempt direct repair, showing a good alternative procedure to tendon grafting. Finally, Ayalon et al. presented cases with intervals of between 2 and 96 weeks after trauma [14]. In their case series, the worst outcomes expressed in terms of TAM were reported when patients were treated between 2 and 3 weeks after trauma. However, they did not correlate these outcomes with the interval between trauma and treatment.

## 4. Discussion

The present report evaluated the functional outcomes of repairs to flexor tendon injuries in zone 1 and jersey finger type 1 performed more than 3 months after trauma, managed in our department. Despite the period of time that had elapsed, in four of our cases, a direct suture or flexor tendon reinsertion was possible in association with other procedures, leading to satisfying functional recovery.

The COVID-19 pandemic has significantly altered the dynamic between patients and healthcare systems. Due to stringent isolation measures and widespread fear of contagion, a substantial number of patients delayed or avoided seeking medical care, even when clinically indicated. This phenomenon has posed new and complex therapeutic challenges for orthopedic surgeons, particularly in the management of delayed presentations of hand flexor tendon injuries. The shift in patient behavior has necessitated a reevaluation of treatment strategies and timelines, especially in cases where early surgical intervention is traditionally considered critical for optimal functional recovery [3,4,5,6,7,8,9,10,11,12,13,14,15,20].

Two months from injury is assumed to be a reasonable cutoff for direct primary repair, as reported by Unsal [4]. This timeframe has been reported by a few surgeons in response to a questionnaire. Moreover, the literature reports unsuccessful results following later tendon repair due to excessive tension of the proximal stump, leading to a severe extension lag of the finger. For this reason, Ejeskär, in 1980, suggested not attempting a direct flexor tendon repair if 3 months had elapsed since the injury [3]. This recommendation was made based on surgical practice in the late 1970s, but great improvements have since been made in flexor tendon repair concerning concepts, techniques, materials, and rehabilitation protocols. Additional further procedures to avoid excessive tension, such as FDP lengthening and Z-plasty of the myotendineous junction, were described for the first time a few years after the release of Ejeskär’s paper [9,10]. The fact that no tendon elongation was performed could be the reason for the poor results obtained by Ejeskär—in which the main drawback was, in fact, extension lag.

In that period, Suzuki also reported very late flexor tendon repair, but the best results were obtained in children, in whom tissue plasticity is definitely greater than that in adults [12].

More recently, Ayalon reported an impressive series of flexor tendon repairs performed more than 3 months after injury [14]. Like the previous authors, he did not describe any associated procedures, even in late repair, stating that repair was possible when vincula caught the proximal stump in proximity to the injury. Only for the FPL did he discuss performing tendon elongation instead of grafting, since the latter consists in a more demanding procedure with unpredictable results compared to those provided by direct repair [16].

The reason why Ayalon did not require tendon elongation in his series, as we did, is unknown [14]. Tendon elongation was required by Sawaya in their report on very late tendon reinsertion [11]. Despite this, the innovations introduced in flexor tendon repair in the last 20 years, such as suture materials, suture techniques, and rehabilitation protocols, greatly influenced the final results observed by comparing Ejeskär’s series [3] to the most recent experiences, such as the present patients and Ayalon’s study [14].

Another critical aspect of successful repair is the integrity of the pulley system. It is imperative that at least two pulleys are patented through to possibly run the tendon. This was also stated by Ayalon when describing the surgical technique [14]. Otherwise, a two-stage reconstruction must be planned.

An alternative procedure for when a gap cannot be overcome by means of direct suture, even with intact pulleys, is represented by tendon grafting. In the literature, tendon grafting is associated with poorer results compared to direct repair. In particular, Samora reviewed the literature that has reported satisfactory or good results, especially when the FDS is uninjured [21]. We note that all of the reported studies were conducted in the 1960s and 1970s. The other aspect to be considered is that tendon grafting is indicated for radial long fingers. When treating an ulnar finger with an intact FDS (as for our patients), direct repair is the first choice when feasible; otherwise, other procedures are to be considered. Moreover, another study reported average TAM of 186° after single-stage reconstruction, which is inferior to the results described here [22]. We note that two-stage reconstructions are feasible nowadays. For example, Coyle reported thirty-five reconstructions with average TAM of 206° [23], whereas Beris et al. operated on twenty-two fingers with the modified Paneva−Holevich technique, obtaining a mean TAM of 189° [24]. This means that two-stage procedures, whether Hunter’s or Paneva−Holevich’s, can also lead to successful results; therefore, two surgical procedures are required and should be proposed in radial fingers or in patients with high functional requirements, and only when an immediate repair or reconstruction is unfeasible. Examples include cases where the tendon gap exceeds 3 cm and adjunctive procedures—such as fractional lengthening, tenolysis, or Z-plasty—would not reasonably allow for direct repair, or cases requiring pulley reconstruction (e.g., when only one pulley is deemed reliable).

An alternative to grafting was proposed by Durand [13]. He performed an emi-FDP transfer from an adjacent finger to repair a tendon lesion. He described the results obtained in a small group of patients treated around 5 months after trauma. They presented FDP injury in either zone 1 or 2, without any other injuries. The study did not present full data regarding functional recovery but reported “nearly full recovery”. This procedure has the advantage of not requiring Z-plasty, and lengthening and can be adopted when flexor tendon elongation is not sufficient. Despite these advantages, an adjacent healthy finger must be dissected, risking postoperative swelling and adhesions.

The case series presented here indicates the possibility of obtaining good functional results when a direct flexor tendon repair is performed even beyond the limit of the 6–9 weeks proposed in the literature. This aligns with results from Ayalon, presenting even more patients repaired after more than 3 months with outstanding results [14]. The discussed cohort of patients of the present paper was small and limited to FDP or FPL injury, while Ayalon’s study also reported on combined FDS and FDP injuries, with less favorable results. Unfortunately, he did not stratify the results by the time since trauma, but he noted that the worst results occurred when the repair took place between weeks 2 and 3 after trauma, probably because of the swelling and inflammatory environment [14].

The results obtained were comparable to those described elsewhere regarding primary flexor tendon repair. Townsend obtained a mean TAM of between 180° and 190° in long finger flexor tendon repair in zone 2 [25]. The DIP joint is the most commonly affected, with persistent stiffness (particularly in extension) being a frequent limitation. In the present cases, an extension lag of 10° to 20° persisted, which was slightly greater than that reported by Townsend [25]. The worst result was obtained following reinsertion of the deep flexor (jersey finger), a finding comparable with the largest case reports on the treatment of this pathology [26,27]. Recovery of DIP joint motion was generally the most limited. This may be related to the degree of joint mobility maintained by the patient prior to surgery, as well as to individual factors such as constitutional laxity and habitual activity. For example, the patient who achieved the greatest DIP flexion was a semi-professional musician who had actively maintained DIP mobility before surgery. Notably, DIP stiffness alone rarely appears to significantly limit overall functional recovery of the hand.

Given the complexity of these procedures and the potential for complications, we recommend that they be performed at specialized hand surgery centers by experienced surgeons in order to possibly limit repairs that may lead to excessive finger flexion, allow proper management of the frequent associated injuries to the phalanges and vascular bundles, and, at the same time, reduce the incidence of postoperative complications. Furthermore, as reported by other authors, it is imperative to treat patients with supple and smooth fingers without swelling or joint stiffness [3,4,5,6,7,8,9,10,11,12,13,14,19,20].

Numerous alternative procedures can be proposed, especially when a competent FDS is present in long fingers. Moreover, it is not always possible to perform a direct delayed repair of the FDP. For these reasons, it is essential to fully inform the patient about the procedures that will be performed, the possible complications, the therapeutic and rehabilitative processes required for tendon repairs, and alternative treatment options such as joint fusion or tenodesis. A clear reconstructive program should be discussed with the patient in order to perform the most appropriate procedure given these requirements.

Based on the above considerations, these procedures should be reserved for young, compliant, and highly motivated patients with high functional demands, such as musicians. Equally important is the presence of a good passive range of motion and the ability to adhere to a full, consistent, and intensive rehabilitation program.

The principal limitations of this study are related to the small sample size, consisting of only four patients, which inherently constrains the statistical power and external validity of the findings. Despite this constraint, the results obtained appear to be superior to those reported in previous literature. This may be attributable to the context of care: a high-volume, subspecialized center with consolidated expertise in hand surgery and access to standardized, protocol-driven rehabilitation pathways. The integration of advanced surgical techniques with early and individualized physiotherapeutic management likely contributed to the favorable results. Nevertheless, the small cohort size introduces a potential selection bias and limits the generalizability of the data. Future multicentric studies with larger populations are warranted to validate these preliminary observations.

## 5. Conclusions

In conclusion, the case series discussed herein demonstrates the possibility of performing direct FPL and FDP tenorrhaphy or reinsertion with good functional recovery, even with a timeframe longer than those proposed in the literature, such as a reasonable delay for primary flexor tendon repair of 6 [4,19] to 12 [3] weeks. In this report, it was necessary to perform associated procedures such as tenolysis, fractional lengthening, or Z-lengthening at the myotendinous junction in all treated patients to close the gap. Further research is required to strengthen these findings and to further examine the time limit for primary flexor tendon repair. Surgical skill and adequate patient counseling and selection are required to achieve valid functional results in these cases.

## Figures and Tables

**Table 1:** Patient characteristics and associated procedures required to perform a direct tendon suture.

Diagnosis/Lesion	Sex	Age	Time Between Trauma and Surgery (Months)	Associated Lesions	Associated Procedure
FDP, little finger	Male	34	4	-	Fractional lengthening at the myotendinous junction
FDP, little finger	Female	34	7	Ulnar collateral nerve lesion	Fractional lengthening at the myotendinous junction
Jersey finger type 1, ring finger	Male	32	3	P2 head fracture	Tenolysis
FPL, thumb	Female	19	8	-	Z-plasty at the myotendinous junction

FDP = flexor digitorum superficialis; FPL = flexor pollicis longus.

**Table 2:** Range of motion of affected finger at last follow-up.

Finger	Lesion	MP	PIP	DIP	TAM	Kapandji Score	Type of Recovery
Little finger	FDP	0–90°	10–110°	10–80°	240°		92% Excellent
Little finger	FDP	0–90°	5–120°	20–40°	225°		77% Good
Ring finger	Jersey finger type 1	0–90°	0–80°	0–10°	180°		69% Acceptable
Thumb	FPL	0–90°	0–45°	/	135°	9	75% Good

MP = metacarpophalangeal; PIP = proximal interphalangeal; DIP = distal interphalangeal; TAM = total active motion; FDP = flexor digitorum profundus; FPL = flexor pollicis longus.

**Table 3:** Main features of the articles considered for this review.

Paper	Intervention Type	Population	Time Between Trauma and Repair	Aims	Methodology Adopted	Key Findings	Gaps in the Research
Suzuki 1976 [12]	Direct repair with buried suture and fascial graft	8	20 days to 4 years	Functional recovery	Loss of flexion and extension	Recovery of nearly full ROM, especially in children; less favorable in adults	Only 3 patients were treated more than 3 months after injury
Ejeskar 1980 [3]	Direct repair with stainless-steel suture	42	2–14 weeks	Functional recovery	TAM evaluation between 6 and 24 months after repair	Authors concluded that even late repair is functionally better than tendon graft (compared with literature results); in late repair, extension lag occurs	Comparison to tendon grafts by other authors; functional results obtained were not divided according to time from trauma
Durand 2010 [13]	Emi-FDP transfer	4	5 months	Functional recovery	ROM	Nearly full recovery except in one patient	Full data were not reported; authors did not mention alternative surgery
Sawaya 2012 [11]	Direct repair of jersey finger type 2 with Z-plasty for FDP of ring finger	1	6 weeks	Functional recovery	TAM evaluation	TAM 220° at 6 months	Case report
Munz 2021 [19]	Direct repair with M-Tang suture	28	15 days (range 4–37)	Functional recovery and complications	Tang criteria and Buck-Gramcko criteria	Excellent or good results in 87% of digits	Authors did not treat cases more than 3 months after injury
Ayalon 2022 [14]	Direct repair with modified Kessler or modified Bunnel + epitendinous suture	46	2–96 weeks (8.5 weeks mean) for finger, 2–17 weeks (5.5 weeks mean) for thumb	Functional recovery	TAM evaluation	Excellent or good results in 73% of cases when FDP only was injured; when both tendons were injured, excellent–good results lowered to 63%	Results divided according to injury site, not time from injury; authors reported worst outcomes in patients treated between two to three weeks after injury

## Data Availability

The original contributions presented in this study are included in the article/Supplementary Materials. Further inquiries can be directed to the corresponding author.

## Supplementary Materials

The following supporting information can be downloaded at https://www.mdpi.com/article/10.3390/jcm14165796/s1, Figure S1: Male, 34 y, FDP, little finger. Figure S2: Female, 34 y, FDP, little finger. Figure S3: Male, 32 y, jersey finger type 1, ring finger. Figure S4 :Female, 19 y, FPL, thumb. Supplementary Videos: Video S1: Male, 34 y, FDP, little finger. Video S2: Female, 34 y, FDP, little finger. Video S3: Male, 32 y, jersey finger type 1, ring finger. Video S4 Female, 19 y, FPL, thumb.
