# Supplementary figures and images for: Direct Flexor Tendon Repair More than 3 Months After Trauma: Clinical Outcomes of Four Consecutive Cases and Scoping Review on Time Limits
Source: J Clin Med. 2025 Aug 16;14(16):5796. doi: 10.3390/jcm14165796 (PMC12387015; doi:10.3390/jcm14165796)

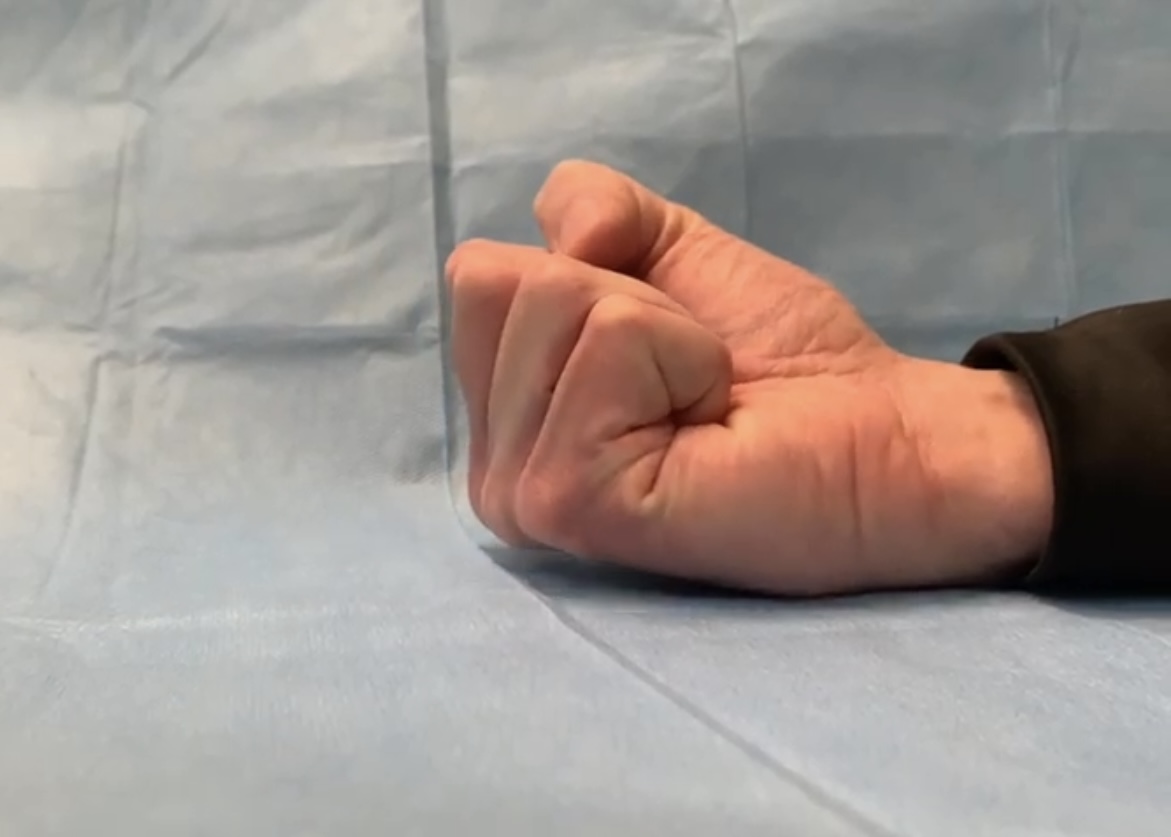

Supplement: Supplementary file 1 [file jcm-14-05796-s001.zip › sup materials/Sup Fig 1.jpg]

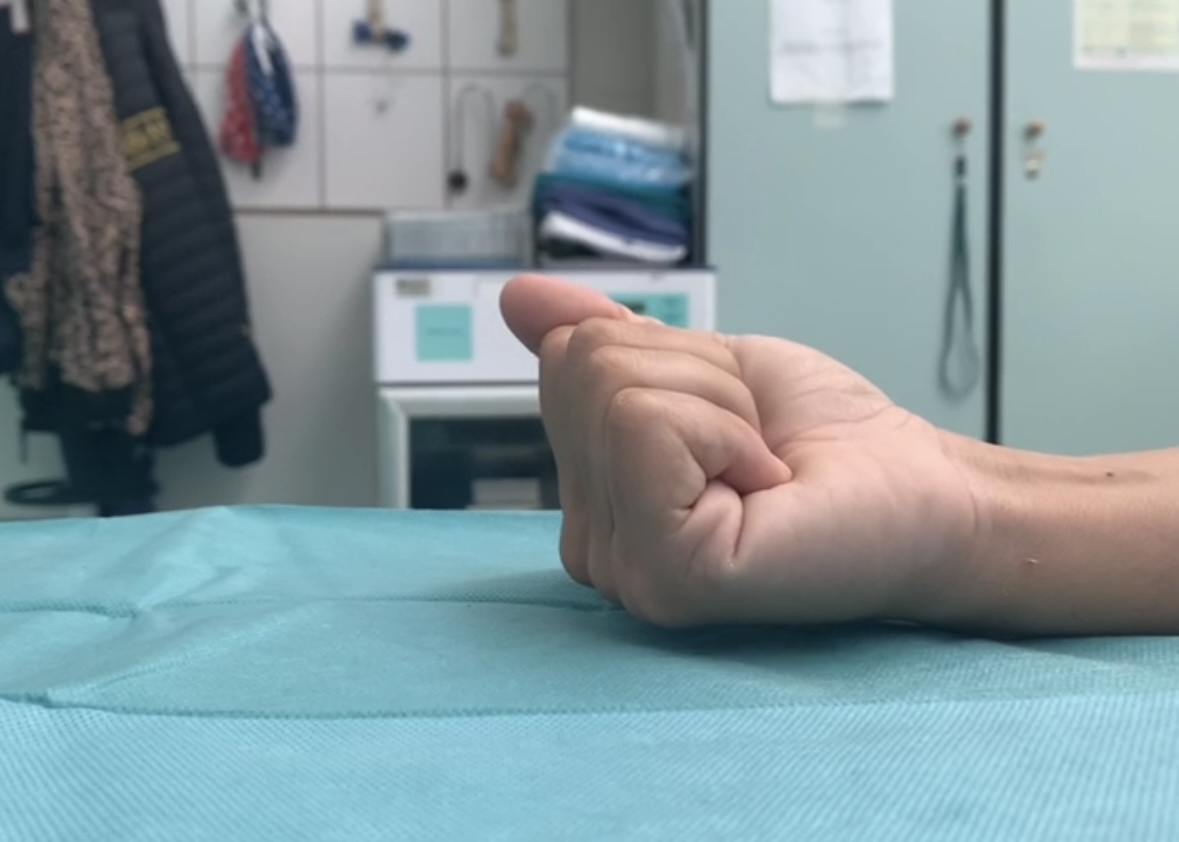

Supplement: Supplementary file 1 [file jcm-14-05796-s001.zip › sup materials/Sup Fig 2.jpg]

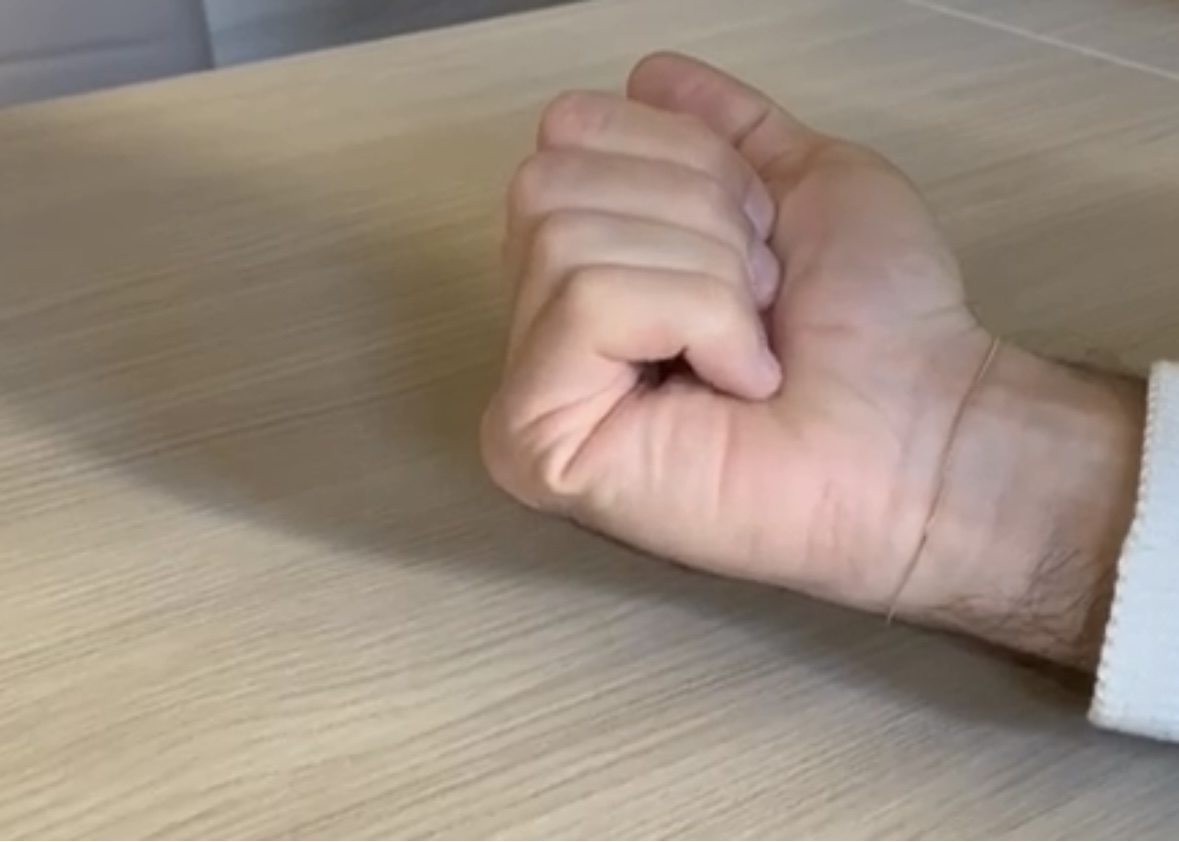

Supplement: Supplementary file 1 [file jcm-14-05796-s001.zip › sup materials/Sup Fig 3.jpg]

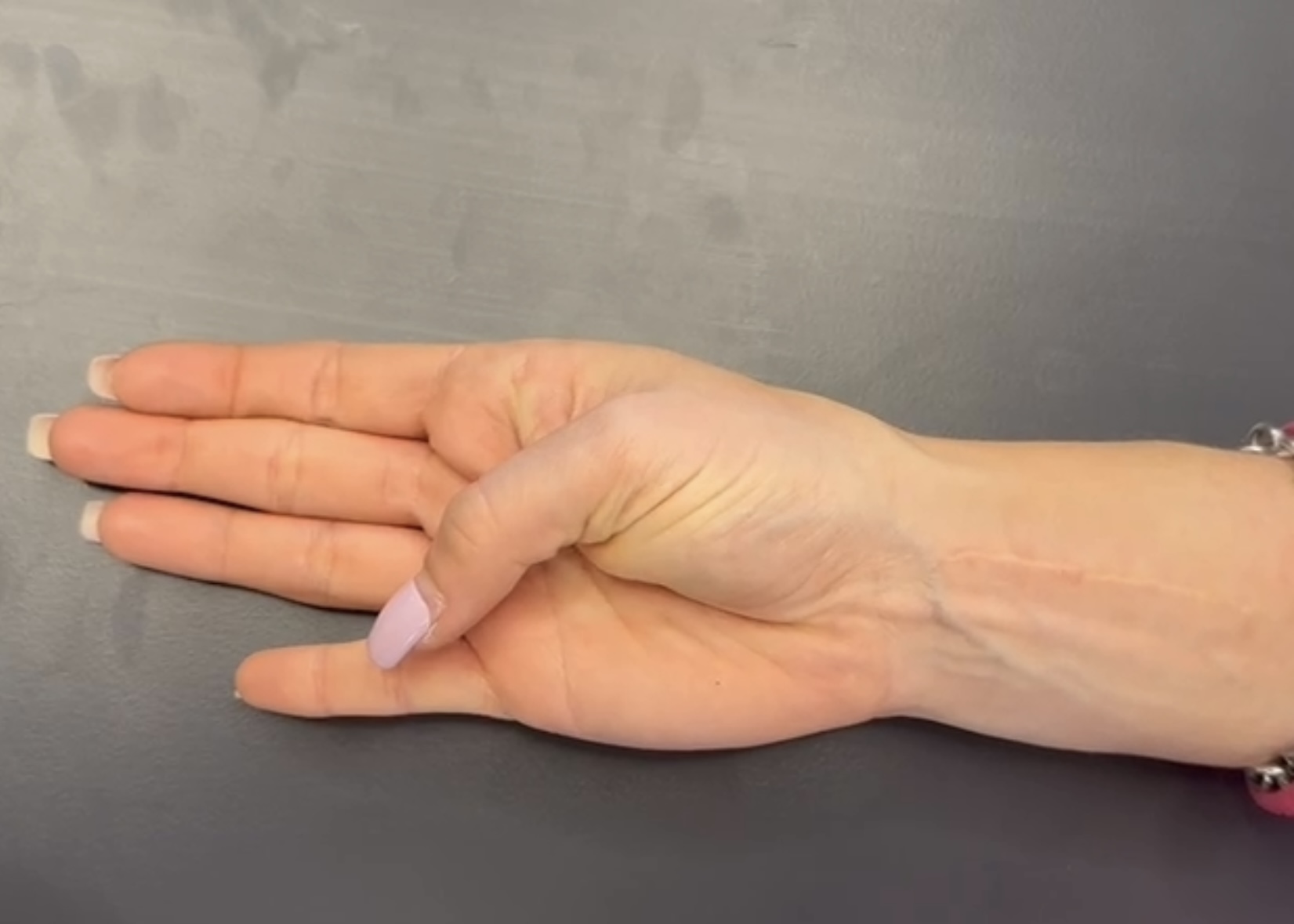

Supplement: Supplementary file 1 [file jcm-14-05796-s001.zip › sup materials/Sup Fig 4.jpg]
